# ONC206 as a low-potency dopamine D_2_ receptor antagonist

**DOI:** 10.1093/noajnl/vdaf185

**Published:** 2025-09-02

**Authors:** Richard Ågren, Kristoffer Sahlholm

**Affiliations:** Department of Neurosurgery, Karolinska University Hospital, Stockholm, Sweden; Department of Physiology and Pharmacology, Karolinska Institutet, Stockholm, Sweden; Wallenberg Centre for Molecular Medicine, Umeå University, Umeå, Sweden; Department of Medical and Translational Biology, Umeå University, Umeå, Sweden; Department of Physiology and Pharmacology, Karolinska Institutet, Stockholm, Sweden

**ONC206 is a small molecule used in trials for treating diffuse midline glioma. The reported mechanisms of action include mitochondrial Clp protease activation and dopamine D_2_ receptor (D_2_R) antagonism. However, there is limited data in the literature on D_2_R engagement. Using a time-resolved functional assay, we assessed ONC206 interactions with D_2_R and observed low-potency antagonism, which was rapidly displaced in competition with dopamine. In vivo plasma concentrations of ONC206 support engagement of D_2_R and highlight this mechanism as a putative contributor to the cytotoxic effect.**

H3 K27M-mutated midline gliomas are classified as WHO grade 4 tumors and remain a clinical challenge. ONC201 is a small-molecule therapeutic of the imipridone class currently in clinical trials. ONC201 shows cytotoxic properties in vitro and clinical efficacy in midline glioma.^1^ Mechanistically, ONC201 acts as a Clp protease activator and low-potency D_2_R antagonist,^2^ the latter characteristic being a feature suggested to contribute to its cytotoxic effect.^3^

ONC206 is an analogue of ONC201 (Figure 1A), with ~5- to 7-fold higher cytotoxic potency in midline glioma cell lines.^4^ Preliminary data from a mammalian cell-based assay suggested a D_2_R antagonist potency for ONC206 of ~320 nM.^5^ Considering the proposed role for D_2_R in mediating cytotoxicity, we investigated ONC206 using a real-time functional D_2_R assay using G-protein-coupled inward rectifier potassium (GIRK) channels. This assay allows for time-resolved characterization of ONC206 binding properties at native and mutant D_2_R.

**Figure 1.**
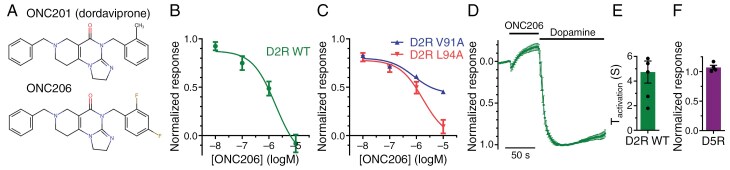
ONC206 is a low-potency D_2_ receptor (D_2_R) antagonist. (A) ONC206 differs by the substitution of the 2-methylphenyl (ONC201) for a 2,4-difluorophenyl moiety. (B, C) Concentration–response relationship of 10 nM–10 µM ONC206 at wildtype (B) and V91A and L94A (C) D_2_R in the presence of 10 nM dopamine. The GIRK currents were normalized to the response evoked by dopamine alone, and sigmoidal functions (Hill slope = 1) were fitted to data. (D) Activation of D_2_R by addition of 100 µM dopamine to previously antagonized receptors (10 µM ONC206). Data are normalized to the maximum response of dopamine. (E) The time constant of the dopamine-induced response is 4.7 ± 0.9 s. (F) ONC206 has no significant effect on the GIRK response evoked by 100 nM dopamine in oocytes co-expressing GIRK channels with D_5_R. Data points represent mean ± SEM from 4 to 6 oocytes.

Human *D_2_R* (wildtype [WT], V91A, and L94A), *dopamine D_5_ receptor* (*D_5_R*)*, GIRK1*, *GIRK4*, and *regulator of G protein signaling 4* (*RGS4*) constructs were linearized using the appropriate restriction enzymes, followed by in vitro transcription.^6^ Oocytes were surgically isolated from the African clawed frog, *Xenopus laevis*, with the approval of the Swedish National Board for Laboratory Animals and the Animal Welfare Ethical Committee in Stockholm. Oocytes were microinjected with 0.2 ng *D_2_R* and 1.6 ng of *RGS4*, or, when used, 25.5 ng *D_5_R*, and 40 pg of each *GIRK1* and *GIRK4* cRNA and incubated for 6 days at 12 °C in an isotonic solution. Electrophysiology recordings were performed using 2-electrode voltage clamp. To increase the potassium channel currents at negative potentials, a high-potassium (25 mM KCl) extracellular buffer was used.^6^ Dopamine was prepared fresh, and ONC206 (MedChemExpress) was dissolved in DMSO and diluted prior to the experiments.

First, ONC206 antagonist potency at the D_2_R WT was evaluated in the presence of a submaximally effective concentration of dopamine (10 nM), yielding a half-maximal inhibitory concentration (IC_50_) of 1.7 µM (corresponding to an inhibition constant [K_i_] of ~1.3 µM; Figure 1B). Application of 10 µM ONC206 to oocytes expressing GIRK channels in the absence of D_2_R did not reveal significant inhibition of the GIRK currents. The role of the D_2_R binding pocket residues valine 91 and leucine 94^2,6^ was evaluated by alanine substitution. Whereas D_2_R L94A mutation did not affect ONC206 antagonist potency, D_2_R V91A lowered the maximal extent of antagonism, suggesting that valine 91 is important for ONC206 binding (Figure 1C), similar to findings reported with ONC201.^2^ Finally, response recovery from ONC206 was assessed by first antagonizing D_2_R with ONC206, followed by a supramaximal application of dopamine, which evoked a rapid response (Figure 1D and E).

Notably, for ONC206 to fully antagonize D_2_R activation, a high concentration of 10 µM is required despite competing against a low D_2_R agonist concentration. Under the assumption of competitive D_2_R antagonism (*K*_*i*_ = 1.3 µM; although allosteric-like effects of imipridones have also been noted^2^), this would suggest a 7-fold higher D_2_R potency of ONC206 compared to that of ONC201 (*K*_*i*_ = 9.3 µM^2^), which correlates well with the ~5- to 7-fold higher cytotoxic potency of ONC206.^2^ However, it should be noted that Clp protease engagement and induction of death receptor 5 are also likely to contribute to the observed cytotoxic potency of ONC206.^7^ Furthermore, the D_5_R has been implicated as a site of action of ONC206.^7^ However, in our hands, ONC206 did not reduce D_5_R-mediated GIRK responses to dopamine^8^ at a concentration of 10 µM (Figure 1F).

Although data on ONC206 concentrations in H3 K27M gliomas are currently the subject of investigation within a Phase I trial,^9^ oral ONC206 doses of 50–150 mg have been shown to result in peak plasma concentrations of ~0.5–1.5 µM.^10^ Given that ONC206 penetrates the blood–brain barrier, this may support the notion that D_2_R antagonism contributes to ONC206 cytotoxicity, in addition to other mechanisms such as Clp protease activation.

## Data Availability

Data will be made available upon reasonable request.
